# Ascorbic and silicic acid application mitigated toxic effects of ozone in mung bean (*Vigna radiata* L. Wilczek) by modulating growth, secondary metabolites, water relations, and grain quality attributes

**DOI:** 10.1002/jsfa.14185

**Published:** 2025-02-19

**Authors:** Naeem Iqbal, Eram Shahzadi, Muhammad Nawaz, Muhammad Shahid, Fahad Khan

**Affiliations:** ^1^ Department of Botany Government College University Faisalabad Faisalabad Pakistan; ^2^ Nuclear Institute for Agriculture and Biology (NIAB) Faisalabad Pakistan; ^3^ Tasmanian Institute of Agriculture (TIA) The University of Tasmania (UTAS) Hobart Australia

**Keywords:** plant biomass, tropospheric ozone stress, plant growth regulators, mung bean

## Abstract

**BACKGROUND:**

Elevated levels of tropospheric ozone (O_3_) pose a significant threat to plant health and productivity. Developing ozone‐tolerant varieties is crucial for mitigating these environmental stresses. This study investigates the effects of ascorbic acid (AA) and silicic acid (SA) treatments on 12 different mung bean varieties under elevated O_3_ conditions.

**RESULTS:**

A controlled pot experiment was conducted with four treatments: ambient O_3_ (40–45 ppb), elevated O_3_ (120 ppb), elevated O_3_ with silicic acid (0.1 mmol L^−1^), and high O_3_ with ascorbic acid (10 mmol L^−1^). High O_3_ stress negatively impacted growth attributes across all mung bean cultivars. However, both AA and SA treatments significantly alleviated O_3_‐induced growth reductions. Under O_3_ stress, osmotic potential, water potential, relative water content, turgor potential, sugars, pod number, amino acids, 100‐seed weight, and grain carbohydrates all decreased. In contrast, antioxidant enzymes (ascorbate peroxidase, peroxidase, catalase, and superoxide dismutase), flavonoids, tannins, and grain protein content increased.

**CONCLUSION:**

The NIAB Mung 20‐21, NIAB Mung 2006, and NIAB Mung varieties exhibited O_3_ resistance. Silicic acid proved to be more effective than ascorbic acid in mitigating O_3_ damage, though a combination of both treatments was the most beneficial for enhancing plant resilience under elevated O_3_ conditions. © 2025 The Author(s). *Journal of the Science of Food and Agriculture* published by John Wiley & Sons Ltd on behalf of Society of Chemical Industry.

## INTRODUCTION

In the context of increased food demand due to an ever‐increasing population, environmental factors that affect plant development and growth are a major source of concern worldwide. The effects of biotic and abiotic stresses on crop productivity are extremely harmful. For a long time, agricultural science specialists have been concerned about the constancy of crop output and how it develops in harsh environments, including elevated levels of ozone (O_3_) in the troposphere.^1^ Volatile organic molecules generated by industry embrace the potential to contribute to O_3_ pollution. During summer, when the temperature is high and UV exposure is significant, they play a substantial role in atmospheric photochemical reactions, producing O_3_ explosions close to the surface.^2^ Surface ozone poses a threat to ecosystems and has a negative effect on the physiology, development, and sustainability of plants. Reactive oxygen species (ROS) are produced when this oxidative pollutant enters a plant through open stomata and initiates a series of processes inside the plant cells.^3^, ^4^, ^5^

ROS and the products of their reactions damage cell membranes and interfere with the metabolism of carbohydrates, lipids, nucleic acids, and proteins, according to numerous studies. Agricultural yield is reduced because it alters the structural characteristics of leaves and eventually causes cell malfunction or death.^6^ Enzymatic (superoxide dismutase (SOD), catalase (CAT), ascorbate peroxidase (APX), and peroxidase (POD)) and non‐enzymatic antioxidants (ascorbic acid, tocopherol, glutathione, flavonoids, and carotenoids), which are the primary components of the plant's natural defense system, are among the compounds that plants produce as a line of defense under stress conditions.^7^, ^8^ In emerging countries like Pakistan, Indonesia, and India, as well as in the peri‐urban and urban regions of major Asian cities, elevated tropospheric O_3_ concentration has been rising repeatedly. Due to ambient air pollution, Pakistan's agricultural production appears to be significantly declining.^9^ As background O_3_ concentration has increased globally, scientists have rigorously monitored the impact of rising O_3_ concentration on diverse agricultural plant species in the subtropical zone.^10^

As an unimpeachable source of protein, which is crucial for human growth and development, mung bean (*Vigna radiata* L.) is a better choice for examination of O_3_ effects,^11^ as it is high in bioactive components such as peptides and polysaccharides^1^ and it is included in crops that are most sensitive to ozone (in terms of yield) like wheat, peas, beans, and onion.^12^ Mung bean production was 122.100 thousand tons in 2018^13^ and its production is estimated to be 1.43 million tonnes from 198 000 ha for rabi season 2023–2024 (Federal Committee on Agriculture, Dawn, October 12, 2023). Pakistan was South Asia's second most renowned producer of mung bean in 2009–2010. Numerous biological and physiological characteristics like seed yield, harvest index, days to maturity, and characteristics related to gas exchange such as transpiration rate, stomatal conductance, net photosynthetic activity, and water‐use efficiency of different crops, including mung bean, have been affected by the high levels of tropospheric O_3_ observed in Pakistan's suburban agricultural regions.^14^, ^15^ Numerous studies have demonstrated the importance of micronutrients in supporting plants to tolerate various stresses.^16^ One such nutrient – silicic acid, a silicon derivative – is rising in popularity^17^ because of its ability to increase resistance to both biotic and abiotic stresses in many plants such as cucumbers^18^ and rice.^19^ Silicic acid, when used sparingly as a foliar spray, boosts crop yield under stress.^20^ While ascorbic acid represents a ROS scavenger, it improves the plants' oxidative defense capability, resulting in more massive development and growth under stress conditions.^21^ The present study was aimed at evaluating the effectiveness of silicic and ascorbic acid foliar applications in mitigating the detrimental impacts of increased O_3_ on mung bean growth, biochemical attributes, water relation attributes, antioxidant enzymes, and yield.

## MATERIALS AND METHODS

Seeds of 12 mung bean varieties (V1 = NM‐28, V2 = NM‐13‐1, V3 = NM‐19‐19, V4 = NM‐20‐21, V5 = NM‐121‐25, V6 = NM‐51, V7 = NM‐54, V8 = NM‐92, V9 = NM‐98, V10 = NM‐2006, V11 = NM‐2011, and V12 = NM‐2016) with diverse parentages used in this investigation were collected from the Nuclear Institute for Agriculture and Biology (NIAB).

The experiment was carried out from April 2021 to July 2021, and seeds were sown under controlled conditions in a glasshouse. The experiment was conducted in an environment with an average temperature of 30 °C and a relative humidity of 49%. The seeds were planted in soil‐filled plastic pots with four treatments and three replicates. Ascorbic acid (10 mmol L^−1^) and silicic acid (0.1 mmol L^−1^) (selected from pretrial experiment, data not published) were sprinkled on the plants 3 weeks after germination for 1 week, together with Tween‐20 (0.1%) as a surfactant. A pressure pump sprayer bottle was used for this purpose. The elevated ozone levels were exposed to plants (120 ppb) for 4 h per day, except for control (O_3_ untreated). The O_3_ generator (AOT‐MD‐500 model, AQUAPURE (Shenzhen) Ozone Technology Co., LTD) was operated for O_3_ generation. Monitoring was performed using a UV photometry O_3_ analyzer (model 0342e). The elevated O_3_ level was maintained for 15 days. Normal air was considered as the control treatment. After 1 week of O_3_ treatment, leaf samples were collected for estimation of growth and biochemical attributes. Grain quality attributes were studied at maturity.

### Number of leaves

The total number of leaves on each plant were counted manually.

### Determination of total leaf area (cm^2^)

The total leaf area was determined using the following formula:^22^

$$\text{Leaf area}\;\left(cm^{2}\right)=\text{Maximum leaf length}\times \text{Maximum leaf width}\times 0.75$$

where 0.75 is the correction factor.

### Shoot and root

Mung bean plants were measured individually for growth characteristics such as shoot and root length.

### Enzymatic antioxidant activity

Leaf samples were collected to estimate enzymatic antioxidant activity. SOD was evaluated using the method executed by Giannopolitis and Ries,^23^ and the reading was recorded at 560 nm. The enzyme extract was processed according to the Chance and Maehly^24^ method, and the activity of CAT enzyme was determined at 240 nm. The mixture of reactions was prepared according to the methodology of Chance and Maehly,^24^ and the absorbance for POD efficiency was measured at 470 nm. The activity of APX was assessed using the methodology of Asada.^25^ Enzyme activity was detected by a drop in absorbance at 290 nm and represented as units/g F.wt.

### Plant–water relationship attributes

#### Leaf water potential (Ψ_w_)

From each treatment, fully expanded and young second leaves from the top of the plants were utilized to evaluate leaf water potential. From 8 a.m. until 10 a.m., measurements were obtained using a Scholander‐type pressure chamber.

#### Leaf osmotic potential (Ψ_s_)

The leaf that was used to determine Ψ_w_ was also used to determine the osmotic potential, as described by Hussain *et al*.^26^ Leaf samples were frozen at 20 °C and then thawed and crushed with a glass rod to collect cell sap. The sap was then sucked using a disposable syringe and was immediately measured with an osmometer (Wescor 5500, Champaign, Illinois) for Ψ_s_ determination.

#### Turgor potential (Ψ_p_)

The difference between Ψ_w_ and Ψ_s_ values was used to compute the turgor potential as reported by Hussain *et al*:^26^

$$\left(\Psi_{p}\right)=\left(\Psi_{w}\right)-\left(\Psi_{s}\right)$$

#### Relative water content

Three plants from each treatment were sampled for flag leaf samples. Each sample was weighed (*w*_F_) and stored for 24 h in a test tube containing distilled water. Tissue paper was then wiped over and used for its turgid weight (*w*_T_). The samples were dried for 72 h at 65 °C, after which their dry weight (*w*_D_) was calculated. Relative water content (RWC) was calculated for each treatment using the following formula:^27^

$$\mathrm{RWC}\;\left(\%\right)=\left[\left(w_{F}-w_{T}\right)/\left(w_{F}-w_{D}\right)\right]\times 100$$

### Reducing and non‐reducing sugars (sugar content)

Dinitrosalicylic acid was used to measure the total reducing sugars in the plant (Miller 1959). Total soluble sugars were measured following.^28^ The difference between soluble and reducing sugars was used to compute non‐reducing sugars.

### Total flavonoid content

A colorimetric approach was implemented to determine the analysis, with quercetin serving as the standard.^29^ 10 g leaf tissue was used to make 200 μL filtrate, which was prepared using 95% methanol extract and 40 mmol L^−1^ phosphate buffer (pH 6.8). After adding 50 μL AlCl_2_ (10%) and 1 mol L^−1^ potassium acetate (50 μL) the mixture was incubated for 40 min at room temperature before absorbance at 415 nm.

### Tannin

After keeping 0.5 g leaf sample in 95% methanol for 48 h in the dark, the supernatant was collected and mixed with 150 μL **of** 100% **Folin–Ciocâlteu** reagent and 1.2 mL of 700 mmol L^**−1**^ sodium carbonate. This mixture was allowed to cool for 1 h at room temperature.^30^ Then, in the above‐prepared sample, 0.1 g **polyvinylpolypyrrolidone** was added; **the mixture was** vortexed and centrifuged at 14 000 × *g*, and the absorbance was measured at 765 nm.

### Number of pods and 100‐seed weight

The mean number of pods per plant was calculated after counting the number of pods from **ten** random plants of each var**iety** from each replicate. The 100‐seed average weight was recorded using **a** digital weight balance after taking 100 seeds from **ten** random plants of each replicate.^15^

### Grain quality attributes

#### Grain protein

The micro‐Kjeldhal technique was used to quantify nitrogen of grains as described by Bremner.^31^ In order to determine the quantity of grain protein (GP), the nitrogen percentage was multiplied by a factor of 6.25.

#### Grain carbohydrates

Determination of starch was performed according to the method of Malik and Srivastava.^32^ 1 mL of the extract was placed in a 25 mL test tube, followed by 10 mL anthrone solution. It was heated for 12 min in boiling water, then cooled and measured absorbance at 625 nm.

#### Free amino acids

Hamilton's^33^ method was used to determine total free amino acids (FAA). For this, 1 mL of the extract used in the antioxidant enzyme activity was combined with 1 mL ninhydrin and 1 mL pyridine. The mixture was heated at 95 °C for 35 min and absorbance was measured spectrophotometrically at 570 nm.

### Statistical analysis

Each experimental unit had three replications during split‐plot analysis. Statistical analysis of the obtained data was performed using Co‐Stat version 6.4.5.1 (**2003;** Cohorts Software, Monterey, CA, USA) to assess the significance of differences among mean values. The correlations and principal component analysis of the mean values of all variables were performed using R Studio.

## RESULTS

**Figure 1:**
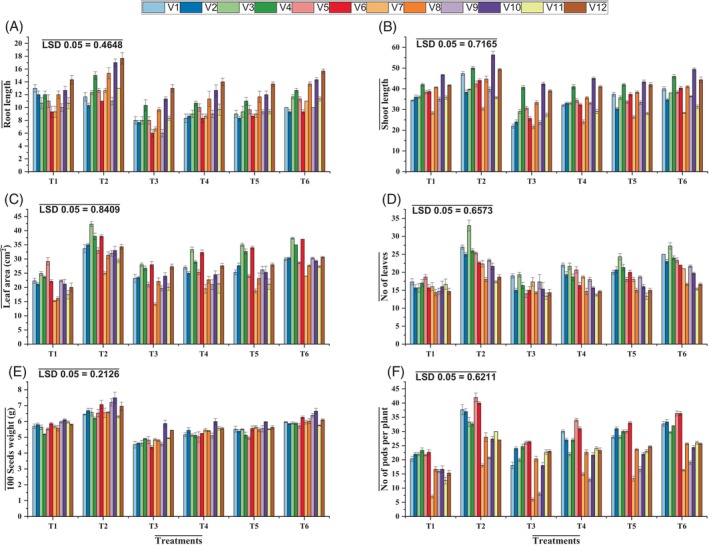
Effects of applying ascorbic acid and silicic acid on mung bean varieties under elevated level of O_3_. Root length (A), shoot length (B), leaf area (C), number of leaves (D), 100‐seed weight (E), and number of pods per plant (F). T1 = ambient ozone level; T2 = control + silicic acid + ascorbic acid; T3 = elevated ozone level 120 ppb; T4 = elevated ozone level + silicic acid; T5 = elevated ozone level + ascorbic acid; T6 = elevated ozone level + silicic acid + ascorbic acid.

The application of O_3_ stress (120 ppb) resulted in a significant reduction in the shoot and root lengths of all mung bean varieties. However, because of genetic diversity among varieties, there was a difference in the rate of decrease among them. Foliar treatment with ascorbic acid significantly (*P* ≤ 0.001) increased the shoot and root lengths of the varieties NIAB Mung‐28 and NIAB Mung‐98. Silicic acid application (0.1 mmol L^−1^) as a foliar spray significantly (*P* ≤ 0.001) increased shoot and root length under O_3_ stress conditions in NIAB Mung‐28, followed by NIAB Mung‐98. It was found that NIAB Mung‐2011 was less affected by silicic acid foliar application (Table 1 and Fig. 1).

**Table 1:** Data from analysis of variance (ANOVA) demonstrating changes in root length, shoot length, leaf area, number of leaver, number of pods per plant, and seed weight by foliar spray of silicic acid and ascorbic acid on 12 mung bean varieties under elevated ozone stress and non‐stress conditions

		Root length (cm)	Shoot length (cm)	Leaf area (cm^2^)	No. of leaves per plant	No. of pods per plant	100‐Seed weight (g)
Main effects	df	MS	MS	MS	MS	MS	MS
Variety (*V*)	11	60.08***	597.69***	280.00***	121.52***	629.39***	1.043***
Treatment (*T*)	5	96.95***	718.21***	777.14***	319.45***	879.93***	15.10***
*V* × *T*	55	1.64***	14.88***	11.34***	9.21***	16.21***	0.29***
Error	144	0.99	2.37	3.26	1.99	1.78	0.21
Total	215						

Abbreviation: df, degree of freedom; MS, Mean square values.

***highly significant at *P*≤0.001.

Both the number of leaves and the leaf area per plant considerably decreased (*P* ≤ 0.01) at elevated O_3_ levels and NIAB Mung‐98 exhibited the maximum decline in the said attributes. In this study, foliar treatments with silicic and ascorbic acids on the NIAB Mung 13‐1 under O_3_ stress were more effective (Table 1 and Fig. 1).

Elevated O_3_ significantly decreased (*P* ≤ 0.001) the weight of 100 seeds and the number of pods per plant. This drastic decline in yield was observed in the mung bean varieties NIAB Mung 13‐1 and NIAB Mung‐51. The varieties NIAB Mung‐51 and NIAB Mung 13‐1 treated with ascorbic acid and silicic acid produced a greater number of pods per plant, and 100‐seed weight increased in NIAB Mung‐51 and NIAB Mung‐28 as compared with the untreated control (Table 1 and Fig. 1).

**Figure 2:**
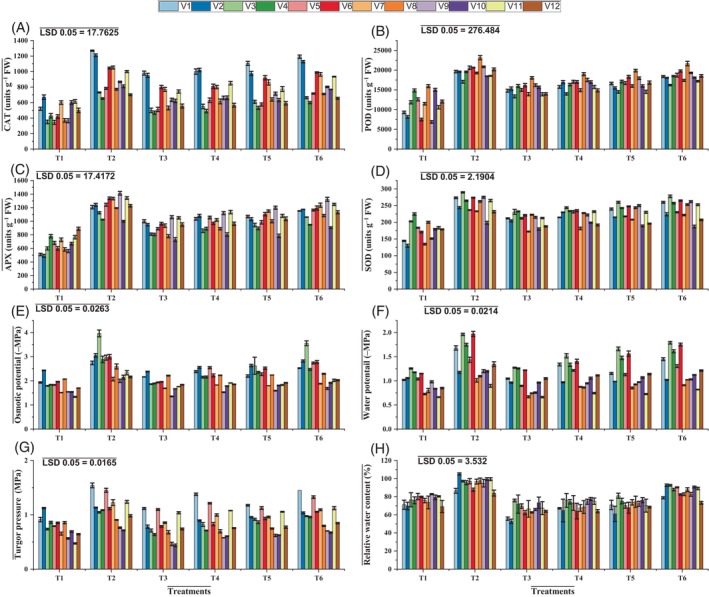
Effects of applying ascorbic acid and silicic acid on mung bean varieties under elevated level of O_3_. CAT (A), POD (B), APX (C), SOD (D), osmotic potential (E), water potential (F), turgor potential (G), and relative water content (H). T1 = ambient ozone level; T2 = control + silicic acid + ascorbic acid; T3 = elevated ozone level 120 ppb; T4 = elevated ozone level + silicic acid; T5 = elevated ozone level + ascorbic acid; T6 = elevated ozone level + silicic acid + ascorbic acid.

Results of the current study revealed that elevated O_3_ significantly (*P* ≤ 0.001) increased the activities of SOD, APX, CAT, and POD in mung bean varieties under investigation. Ascorbic acid treatment significantly improved (*P* ≤ 0.001) activities of different antioxidant enzymes, namely SOD (NIAB Mung 13‐1), POD (NIAB Mung‐98), CAT (NIAB Mung‐51), and APX (NIAB Mung 13‐1) in different varieties. Silicic acid application also augmented the activities of enzymatic antioxidants, namely SOD (NIAB Mung‐28), POD (NIAB Mung‐51), CAT (NIAB Mung‐51), and APX (NIAB Mung‐98) in the different varieties under investigation (Table 2 and Fig. 2).

**Table 2:** Analysis of variance (ANOVA) data demonstrating changes in catalase (CAT), peroxidase (POD), ascorbate peroxidase (APX), superoxide dismutase (SOD), osmotic potential (OP), water potential (WP), turgor potential (TP), and relative water content (RWC) by foliar spraying of silicic acid and ascorbic acid on 12 mung bean varieties under elevated O_3_ stress and non‐stress conditions

		CAT	POD	APX	SOD	OP (−MPa)	WP (−MPa)	TP (MPa)	RWC (%)
Main effects	df	MS	MS	MS	MS	MS	MS	MS	MS
Variety (*V*)	11	495 454.3 ***	29 946 483***	147 997.9 ***	7292.742***	0.78317***	1.27230***	0.675469***	307.8903***
Treatment (*T*)	5	788 076.7***	3.08812e8***	1 412 117.4***	28 511.40***	1.28143***	1.02560***	0.607309***	4350.770***
*V* × *T*	55	13 211.39***	5 559 703***	18 890.636***	638.0869***	0.02299***	0.02798***	0.037693***	52.20175***
Error	144	1453.643	352 197.6	1397.6713	22.10572	0.00318	0.00210	0.00125	57.48307
Total	215								

Abbreviation: df, degree of freedom; MS, Mean square values.

***highly significant at *P*≤0.001.

Elevated O_3_ stress caused a significant (*P* ≤ 0.001) decrease in the water potential of mung bean varieties. A significant (*P* ≤ 0.001) difference among all varieties was found regarding water potential under elevated O_3_ stress. The variety NIAB Mung‐51 had a greater reduction in water potential, but the variety NIAB Mung‐2016 was found to be superior in this regard. This variable was significantly influenced (*P* ≤ 0.001) by foliar treatment with both ascorbic and silicic acids. Plants treated with 10 mmol L^−1^ ascorbic acid and 0.1 mmol L^−1^ silicic acid under O_3_ stress, for example, generated several‐fold increases in water potential of NIAB Mung 13‐1 and NIAB Mung‐51, respectively (Table 2 and Fig. 2). The results revealed a significant (*P* ≤ 0.001) reduction in the osmotic potential of mung bean varieties. NIAB Mung‐51 demonstrated a concentration‐dependent decrease in osmotic potential under O_3_ stress conditions.

Exogenous foliar treatments had a significant (*P* ≤ 0.001) effect on the osmotic potential of mung bean plants exposed to elevated levels of O_3_. Ascorbic and silicic acids displayed several‐fold increases in osmotic potential in NIAB Mung‐98 (Table 2 and Fig. 2). The turgor potential dropped even more markedly (*P* ≤ 0.001) in NIAB Mung‐98 under O_3_ stress. Foliar treatment with ascorbic and silicic acids significantly enhanced turgor potential in NIAB Mung‐98 and NIAB Mung 13‐1, respectively. Under O_3_ stress, the overall RWC exhibited a non‐significant outcome, whereas NIAB Mung 13‐1 showed a greater reduction. Exogenous applications of ascorbic and silicic acid significantly increased the RWC of NIAB Mung‐92 and NIAB Mung‐28 under O_3_ stress (Table 2 and Fig. 2).

**Figure 3:**
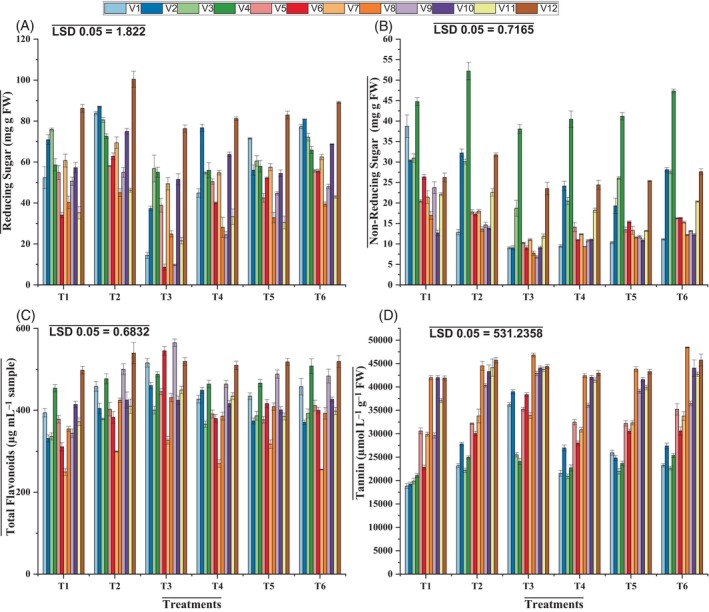
Effects of applying ascorbic acid and silicic acid on reducing sugar (A), non‐reducing sugar (B), total flavonoids (C), and tannin (D) in mung bean varieties under elevated O_3_. T1 = ambient ozone level; T2 = control + silicic acid + ascorbic acid; T3 = elevated ozone level 120 ppb; T4 = elevated ozone level + silicic acid; T5 = elevated ozone level + ascorbic acid; T6 = elevated ozone level + silicic acid + ascorbic acid.

The findings of this study demonstrated that both reducing and non‐reducing sugars decreased significantly (*P* ≤ 0.001). The elevated O_3_ level (120 ppb) caused a drastic decrease in both reducing and non‐reducing sugars in NIAB Mung‐98 and NIAB Mung‐28. In varieties NIAB Mung‐51 and NIAB Mung 13‐1, the foliar treatment with ascorbic and silicic acid resulted in a significant increase (*P* ≤ 0.001) in reducing and non‐reducing sugars under O_3_ stress (Table 3 and Fig. 3).

**Table 3:** Analysis of variance (ANOVA) demonstrating changes in reducing and non‐reducing sugar, total flavonoids, and tannin by foliar spray of silicic acid and ascorbic acid on 12 mung bean varieties under elevated O_3_ stress and non‐stress conditions

		Reducing sugar (mg g^−1^ FW)	Non‐reducing sugar (mg g^−1^ FW)	Total Flavonoids	Tannin (μmol L^−1^ g^−1^ FW)
Main effects	df	MS	MS	MS	MS
Variety (*V*)	11	4303.84***	1520.10***	61 569.20***	1.22698e9***
Treatment (*T*)	5	4516.28***	733.36***	32 807.70***	2.70105e8***
*V* × *T*	55	205.89***	42.50***	2449.80***	17 458 148***
Error	144	15.30	2.15	349.99***	1 300 231.1***
Total	215				

Abbreviation: df, degree of freedom; FW, fresh weight; MS, Mean square values.

***highly significant at *P*≤0.001.

Total flavonoid (TF) and tannin contents in all mung bean varieties significantly increased (*P* ≤ 0.001) under elevated O_3_ levels. Ascorbic and silicic acid applied topically significantly (*P* ≤ 0.001) boosted the flavonoids and tannin levels. Overall, NIAB Mung‐51 was more sensitive to O_3_ stress for flavonoid content and NIAB Mung 13‐1 for tannin content. Application of ascorbic acid proved to be more effective in NIAB Mung 13‐1 for flavonoids and tannins, whereas silicic acid showed a more positive response in NIAB Mung‐98 and NIAB Mung‐28 for flavonoid and tannin contents (Table 3 and Fig. 3).

**Figure 4:**
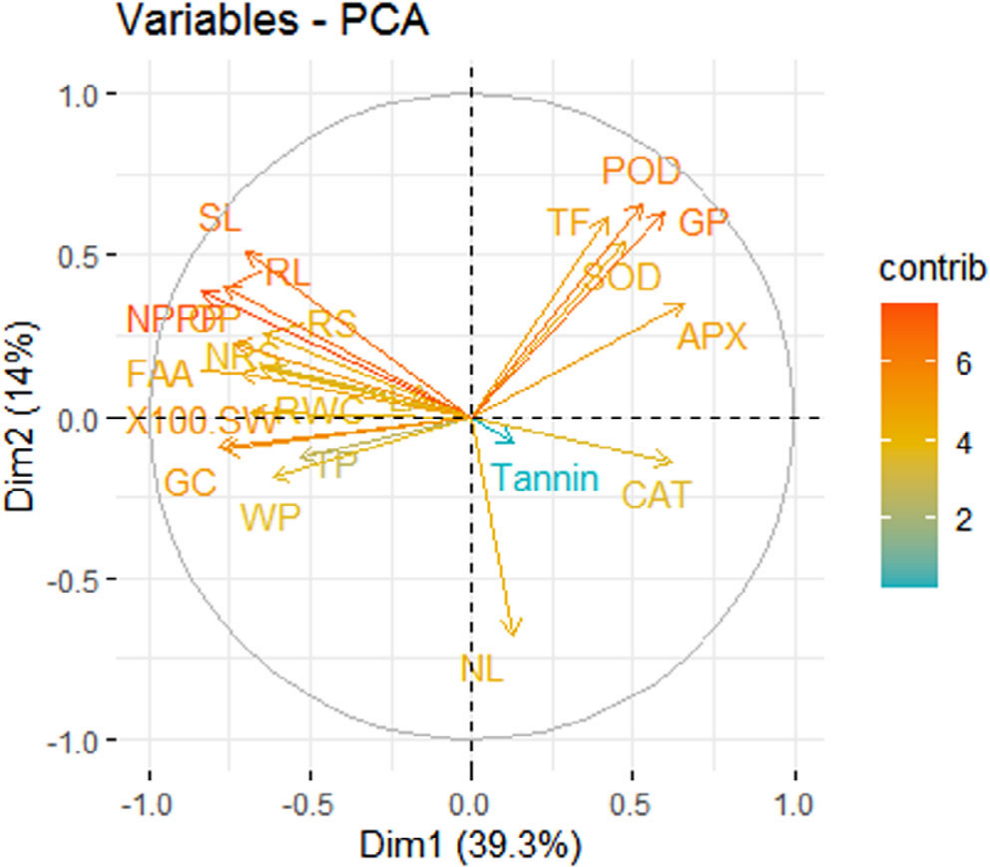
Principal component analysis showing the association among growth, antioxidants, water relation properties, biochemical parameters, and yield attributes in O_3_‐stressed mung bean plants treated with ascorbic acid and silicic acid. 100 SW, 100‐seed weight; APX, ascorbate peroxidase; CAT, catalase; FAA, free amino acids; GC, grain carbohydrates; GP, grain protein; LA, leaf area; NL, number of leaves; NPPP, number of pods per plant; NRS, non‐reducing sugar; OP, osmotic potential; POD, peroxidase; RL, root length; RS; reducing sugar; RWC, relative water content; SL, shoot length; SOD, superoxide dismutase; TF, total flavonoids; TP, turgor potential; WP, water potential.

**Figure 5:**
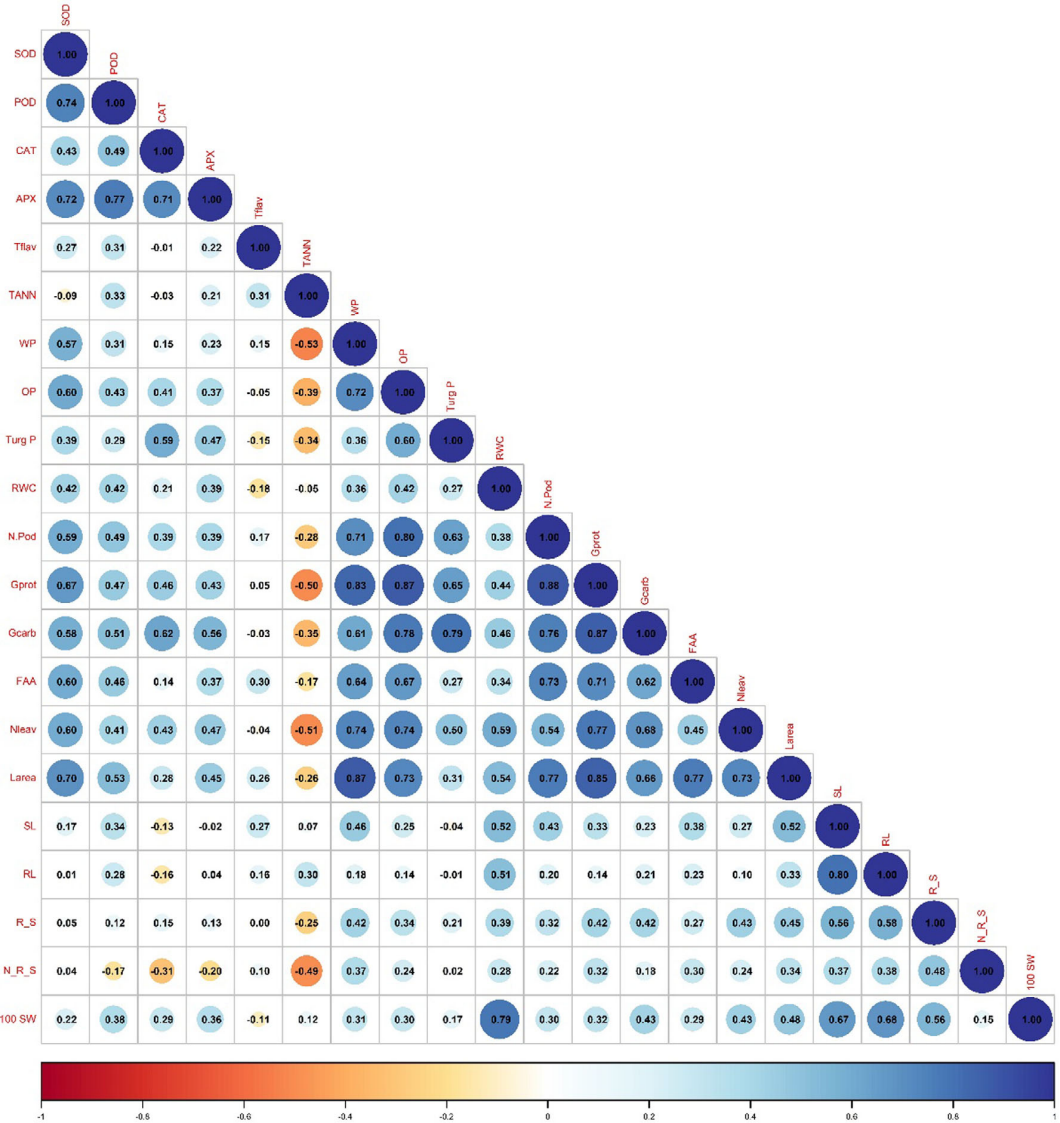
Pearson correlation among the growth, antioxidants, water relation properties, biochemical parameters, and yield attributes of mung bean treated with ascorbic acid and silicic acid under O_3_ stress. 100 SW, 100‐seed weight; APX, ascorbate peroxidase; CAT, catalase; FAA, free amino acids; GC, grain carbohydrates; GP, grain protein; LA, leaf area; NL, number of leaves; NPPP, number of pods per plant; NRS, non‐reducing sugar; OP, osmotic potential; POD, peroxidase; RL, root length; RS; reducing sugar; RWC, relative water content; SL, shoot length; SOD, superoxide dismutase; TANN, tannin; TF, total flavonoids; TP, turgor potential; WP, water potential.

Elevated levels of O_3_ significantly (*P* ≤ 0.001) enhanced GP in all varieties, particularly in the variety NIAB Mung‐51, whereas both the treatments used in this study significantly (*P* ≤ 0.001) boosted GP in NIAB Mung 13‐1 and NIAB Mung‐51. O_3_ treatment had a detrimental effect (*P* ≤ 0.001) on grain carbohydrates in mung bean varieties. Both the applied treatments significantly (*P* ≤ 0.001) enhanced grain carbohydrates in NIAB Mung‐51. A significant (*P* ≤ 0.001) decrease in FAA of mung bean plants was observed under elevated levels of O_3_. NIAB Mung‐98 showed the maximum reduction in FAA under O_3_ stress. Treatments with foliar ascorbic and silicic acids increased maximal FAA in NIAB Mung 13‐1 and NIAB Mung‐98 (Tables 4 and 5). Principal component analysis showed that positive correlations were present among POD, GP, TF, SOD, APX, CAT, number of leaves, and tannin, but a negative correlation of these attributes was found with shoot length, root length, leaf area, number of pods per plant, reducing sugar, non‐reducing sugar, FAA, grain carbohydrates, water potential, osmotic potential, turgor potential, RWC, and 100‐seed weight (Fig. 4). However, POD, GP, TF, SOD, and APX are closely positively correlated, whereas shoot length is closely correlated with root length, leaf area, number of pods per plant, reducing sugar, non‐reducing sugar, FAA, and osmotic potential. Among the extracted components, the major contribution was Dim1 (39.3%), followed by Dim2 (14%), with a cumulative contribution of 53.3%. The same trend is depicted in the Pearson correlation plot (Fig. 5).

**Table 4:** Analysis of variance (ANOVA) demonstrating changes in grain protein (GP), grain carbohydrates (GC), and free amino acids (FAA) by foliar spraying of ascorbic acid and silicic acid on 12 mung bean varieties under elevated O_3_ stress and non‐stress conditions

		GP	GC (mg g^−1^)	FAA (mg g^−1^)
Main effects	df	MS	MS	MS
Variety (*V*)	11	13.43***	9.40***	0.26***
Treatment (*T*)	5	144.20***	0.00***	0.27***
*V* × *T*	55	2.53***	4.48***	0.02***
Error	144	0.36	1.07	9.37
Total	215			

Abbreviation: df, degree of freedom; MS, Mean square values.

***highly significant at *P*≤0.001.

**Table 5:** Effect of elevated O_3_ levels (120 ppb) on grain proteins, grain carbohydrates, and free amino acid content of mung bean varieties after foliar spraying with ascorbic acid and silicic acid

	Grain protein						Grain carbohydrates (mg g^−1^)						Free amino acid (mg g^−1^)					
	T1	T2	T3	T4	T5	T6	T1	T2	T3	T4	T5	T6	T1	T2	T3	T4	T5	T6
NIAB Mung‐28	10.02 ± 0.32	11.87 ± 0.21	4.23 ± 0.27	8.56 ± 0.19	9.29 ± 0.49	10.88 ± 0.23	0.07 ± 0.001	0.10 ± 0.00	0.08 ± 0.00	0.09 ± 0.00	0.08 ± 0.00	0.09 ± 0.00	0.54 ± 0.01	0.54 ± 0.01	0.35 ± 0.01	0.44 ± 0.01	0.37 ± 0.01676	0.47 ± 0.01117
NIAB Mung‐13‐1	9.63 ± 0.42	12.57 ± 0.47	3.29 ± 0.43	9.27 ± 0.60	8.04 ± 0.55	11.12 ± 0.22	0.07 ± 0.00	0.10 ± 0.00	0.07 ± 0.00	0.08 ± 0.00	0.08 ± 0.00	0.09 ± 0.00	0.51 ± 0.02	0.75 ± 0.01	0.57 ± 0.02	0.62 ± 0.02	0.68 ± 0.01076	0.7 ± 0.00806
NIAB Mung‐19‐19	10.77 ± 0.13	9.35 ± 0.22	5.75 ± 0.22	6.44 ± 0.38	7.02 ± 0.51	8.28 ± 0.22	0.07 ± 0.00	0.09 ± 0.00	0.07 ± 0.00	0.07 ± 0.00	0.08 ± 0.00	0.08 ± 0.00	0.47 ± 0.02	0.82 ± 0.01	0.63 ± 0.02	0.65 ± 0.00	0.69 ± 0.00461	0.72 ± 0.01936
NIAB Mung‐20‐21	10.65 ± 0.38	12.347 ± 0.451	9.02 ± 0.33	9.35 ± 0.63	9.71 ± 0.20	11.16 ± 0.11	0.08 ± 0.0008	0.09 ± 0.00	0.06 ± 0.00	0.07 ± 0.00	0.07 ± 0.00	0.08 ± 0.00	0.67 ± 0.02	0.69 ± 0.02	0.47 ± 0.01	0.53 ± 0.03	0.56 ± 0.01023	0.61 ± 0.01505
NIAB Mung‐121‐25	11.08 ± 0.34	11.32 ± 0.07	5.56 ± 0.36	8.73 ± 0.36	7.42 ± 0.25	9.92 ± 0.24	0.07 ± 0.00	0.09 ± 0.00	0.07 ± 0.0	0.08 ± 0.00	0.07 ± 0.00	0.08 ± 0.00	0.39 ± 0.02	0.73 ± 0.01	0.53 ± 0.01	0.61 ± 0.02	0.58 ± 0.00869	0.69 ± 0.00164
NIAB Mung‐51	10.12 ± 0.18	10.12 ± 0.29	3.04 ± 0.35	7.73 ± 0.27	8.42 ± 0.23	9.25 ± 0.06	0.07 ± 0.00	0.09 ± 0.00	0.07 ± 0.00	0.07 ± 0.00	0.08 ± 0.00	0.08 ± 0.00	0.61 ± 0.01	0.82 ± 0.01	0.61 ± 0.01	0.63 ± 0.01	0.68 ± 0.00919	0.71 ± 0.0087
NIAB Mung‐54	11.15 ± 0.36	12.05 ± 0.24	6.50 ± 0.34	8.79 ± 0.36	10.08 ± 0.22	10.95 ± 0.22	0.06 ± 0.00	0.09 ± 0.00	0.06 ± 0.00	0.07 ± 0.00	0.06 ± 0.00	0.08 ± 0.00	0.32 ± 0.00	0.36 ± 0.01	0.05 ± 0.01	0.30 ± 0.00	0.22 ± 0.04879	0.32 ± 0.00696
NIAB Mung‐92	9.41 ± 0.41	10.37 ± 0.23	5.79 ± 0.19	7.67 ± 0.38	8.73 ± 0.37	9.76 ± 0.08	0.06 ± 0.00	0.09 ± 0.00	0.07 ± 0.00	0.07 ± 0.00	0.07 ± 0.00	0.08 ± 0.00	0.38 ± 0.02	0.69 ± 0.01	0.51 ± 0.01	0.53 ± 0.01	0.56 ± 0.01439	0.61 ± 0.01223
NIAB Mung‐98	11.31 ± 0.17	11.30 ± 0.11	4.46 ± 0.36	8.31 ± 0.38	8.91 ± 0.31	10.03 ± 0.21	0.06 ± 0.00	0.08 ± 0.00	0.04 ± 0.00	0.06 ± 0.00	0.07 ± 0.00	0.07 ± 0.00	0.29 ± 0.02	0.76 ± 0.01	0.34 ± 0.04	0.48 ± 0.01	0.57 ± 0.04917	0.71 ± 0.00467
NIAB Mung‐2006	12.35 ± 0.35	11.41 ± 0.39	7.96 ± 0.52	9.23 ± 0.17	8.44 ± 0.59	10.14 ± 0.16	0.06 ± 0.00	0.07 ± 0.00	0.05 ± 0.00	0.06 ± 0.00	0.06 ± 0.00	0.07 ± 0.00	0.49 ± 0.03	0.49 ± 0.01	0.36 ± 0.04	0.40 ± 0.01	0.41 ± 0.02362	0.46 ± 0.006
NIAB Mung‐2011	9.25 ± 0.35	10.82 ± 0.33	4.84 ± 0.47	8.23 ± 0.38	6.62 ± 0.38	9.47 ± 0.20	0.05 ± 0.00	0.08 ± 0.00	0.06 ± 0.00	0.07 ± 0.00	0.06 ± 0.00	0.07 ± 0.00	0.05 ± 0.01	0.58 ± 0.02	0.45 ± 0.03	0.48 ± 0.01	0.45 ± 0.00897	0.52 ± 0.00693
NIAB Mung‐2016	10.99 ± 0.36	12.43 ± 0.43	8.77 ± 0.32	9.65 ± 0.15	10.02 ± 0.21	10.92 ± 0.19	0.06 ± 0.00	0.09 ± 0.00	0.06 ± 0.0	0.07 ± 0.00	0.07 ± 0.00	0.08 ± 0.00	0.46 ± 0.02	0.69 ± 0.01	0.54 ± 0.01	0.57 ± 0.01	0.59 ± 0.00362	0.64 ± 0.00557

## DISCUSSION

The results of this study revealed that altered assimilate partitioning across various plant portions may be the reason for significant reductions in plant development. Because of the uneven distribution of assimilates, plants had fewer and smaller leaves, which hindered shoot and root growth.^34^, ^35^ It may be possible to evaluate whether silicic acid promotes the growth and production of biomass in plants because silicon plays a role in cell replication and extension, as well as in improvements in photosynthesis and light absorption,^36^ as demonstrated in the present work. In addition, it has been discovered that ascorbic acid functions as a cofactor for dioxygenases during the synthesis of auxins (indole‐3‐acetic acid) and gibberellins, which promotes greater plant development.^37^ Ascorbic acid application enhances plant yield and development under normal and stressed conditions as it improves the antioxidant defense system and neutralizes lipid peroxidation of membranes.^35^

In mung bean plants under high levels of O_3_ stress, POD, CAT, SOD, and APX activity was significantly higher, which led to a drop in cellular levels of ROS and the elimination of oxidative stress‐related negative consequences.^38^, ^39^ In the current study, foliar administration of ascorbic acid led to a significant increase in antioxidant enzyme activity. These findings substantiate recent research in which ascorbic acid enhanced the activity of SOD, CAT, POD, and APX enzymes in wheat.^40^ Furthermore, it was found^41^ that exogenous silicon can improve ROS scavenging by influencing antioxidant enzyme activity in wheat (*Triticum aestivum*) and similar findings have been reported recently.^42^, ^43^

The findings of the current research revealed a significant decrease in plant water relation attributes because elevated O_3_ may modify root architecture, limiting root water flow, and, as a result, water availability to above‐ground sections of the target plant.^1^ However, Bybordi^44^ reported that foliar ascorbic acid treatment might help improve plant water status by reducing transpiration water loss, preserving membrane integrity, and controlling osmotic adjustment. Plant defense mechanisms triggered by silicon include a series of signaling cascades that result in physiological and biochemical alterations.^45^ The effect of silicon on root water absorption was studied by Zhu *et al*.,^46^ who reported that root hydraulic conductivity is a measure of a plant's capacity to absorb water. Silicon treatment has recently been shown to improve root hydraulic conductivity in sorghum. Under stress conditions, silicon spray was found to be involved in the modulation of root hydraulic conductivity by upregulating aquaporin gene expression by concentrating potassium inside the xylem sap.^47^

It is assumed that increased mobilization resulted from a decrease in carbohydrate content (reducing and non‐reducing) in the presence of elevated O_3_.^48^ Foliar treatment with ascorbic acid increased sugar deposition by acting as an activator of carbohydrate.^49^ Silicon application tends to allocate more polysaccharides for grain formation compared with total dry matter. Silicon improved stressed flax plants by increasing endogenous phytohormone levels.^50^ The oxidation of amino acids by elevated levels of O_3_ can change the nutritional and metabolic value of grains, although few studies have examined this (e.g., Martin *et al*.).^51^ Additionally, the stimulatory effect of stress on the synthesis of FAA in different mung bean varieties was significantly increased by ascorbic acid application. The breakdown of protein into FAA, which serve as osmoprotectants in the mung bean varieties under study, is probably what led to tolerance against elevated O_3_ stress in the present study. Using silicon foliar treatment, Laane^20^ reported similar results.

With an increase in O_3_ stress, all the investigated mung bean varieties' yield characteristics were noticeably reduced. Increased O_3_ stress, according to Burkey *et al*.,^52^ shortened plant development time due to decreased vegetative biomass production and reproductive growth, which decreased 100‐seed weight and pod number. It is well known that rising O_3_ levels in the atmosphere hinder plant growth and reproduction;^53^ consequently, crop quality and yield decline. Crop yield has been reported to increase when drought‐stressed crops like maize, flax, and wheat are treated with exogenous ascorbic acid.^54^ According to the study conducted by Arshad,^14^ silicon treatment improved rice growth and development, which raised the component of plant yield and decreased elevated O_3_ stress.

Polyphenols, such as flavonoids and tannins, have a significant impact on plant–environment relationships. As reported in a previous study by Akram *et al*.,^55^ higher levels of tannins and flavonoids in the mung bean varieties were found in the current study. TF and tannins, which have a significant structural role in plants and are closely associated with antioxidant activity, are the main bioactive components.^56^, ^57^ The activation of non‐enzymatic antioxidants may account for the rise in polyphenols caused by the foliar application of ascorbic acid.^58^ Similar to what was observed in the current investigation, silicon treatment enhanced secondary metabolism by increasing the generation of flavonoids and tannins.^46^

It might be inferred from the findings of the present study that, under O_3_ stress, all sensitive mung bean varieties experienced significant decreases in their osmotic potential, water potential, RWC, turgor potential, reducing sugar, non‐reducing sugar, number of pods per plant, total FAA, 100‐seed weight, and grain carbohydrates. The results reveal that the varieties NIAB Mung‐2021 and NIAB Mung‐2006 are resistant to O_3_ and that the application of silicic acid is a more effective than ascorbic acid to reduce elevated O_3_ damaging effects. All of this is explained by the interaction of ascorbic acid and silicic acid at the primary and secondary metabolism levels in the tested mung bean varieties to deal with elevated O_3_ stress; it also reflects the genetic variation among the varieties that manipulates all the attributes at the back end.

## CONCLUSIONS

All 12 mung bean varieties under O_3_ stress showed a decline in growth, water relation characteristics, and yield. Among the varieties studied, NM‐20‐21, NM‐2006, and NM‐2016 showed greater growth under O_3_ stress, but NM‐28, NM‐13‐1, NM‐51, and NM‐98 were found to be sensitive to O_3_ stress. All growth traits were less reduced in the O_3_‐tolerant mung bean varieties. In comparison with sensitive varieties, the O_3_ stress typically resulted in a significant change in the activities of SOD, POD, CAT, APX, and all other growth parameters in tolerant ones. To lessen the negative effects of O_3_ on mung bean, a combination of silicic acid and ascorbic acid application was the most effective.

## CONFLICT OF INTEREST

The authors declare that there are no potential conflicts of interest.

## AUTHOR CONTRIBUTIONS

Naeem Iqbal, Muhammad Nawaz, Fahad Khan, and Eram Shahzadi conceived and designed the study. Naeem Iqbal, Muhammad Shahid, and Muhammad Nawaz executed the experiment and compiled the data. Muhammad Shahid, Naeem Iqbal, and Eram Shahzadi helped in sample collection and chemical analysis. Naeem Iqbal and Fahad Khan statistically analyzed the data and helped in chemical analysis. Eram Shahzadi Fahad Khan wrote the manuscript. Muhammad Shahid and Muhammad Nawaz critically edited and revised the manuscript.

## Data Availability

The data that support the findings of this study are available from the corresponding author upon reasonable request.
